# Molecularly Imprinted Polymer-Modified Microneedle Sensor for the Detection of Imidacloprid Pesticides in Food Samples

**DOI:** 10.3390/s22218492

**Published:** 2022-11-04

**Authors:** Samuel M. Mugo, Weihao Lu, Scott V. Robertson

**Affiliations:** Department of Physical Sciences, MacEwan University, Edmonton, AB T5J4S2, Canada

**Keywords:** neonicotinoid microneedle sensor, electrochemical sensing, molecularly imprinted polymers, polyaniline

## Abstract

A portable, molecularly imprinted polymer (MIP)-based microneedle (MN) sensor for the electrochemical detection of imidacloprid (IDP) has been demonstrated. The MN sensor was fabricated via layer-by-layer (LbL) in-tube coating using a carbon nanotube (CNT)/cellulose nanocrystal (CNC) composite, and an IDP-imprinted polyaniline layer co-polymerized with imidazole-functionalized CNCs (PANI-co-CNC-Im) as the biomimetic receptor film. The sensor, termed MIP@CNT/CNC MN, was analyzed using both cyclic voltammetry (CV) and differential pulse voltammetry (DPV) and showed excellent electrochemical performance for the detection of IDP. The CV detection range for IDP was 2.0–99 µM, with limits of detection (LOD) of 0.35 µM, while the DPV detection range was 0.20–92 µM with an LOD of 0.06 µM. Additionally, the MIP@CNT/CNC MN sensor showed excellent reusability and could be used up to nine times with a 1.4 % relative standard deviation (% RSD) between uses. Lastly, the MIP@CNT/CNC MN sensor successfully demonstrated the quantification of IDP in a honey sample.

## 1. Introduction

Due to the rise in the agricultural output over the past few decades, pesticide usage has substantially grown. Imidacloprid (IDP) is a popular neonicotinoid pesticide that is accessible in over 120 countries, and which is effective on over 140 crops, including rice, sugar, and vegetables [1,2]. However, the excessive overuse of IDP results in environmental contamination [3], its accumulation in several agricultural goods, and long-term concerns for human health [2,4]. In recent decades, the bioaccumulation of neonicotinoids and its associated risks to human health has garnered increased attention. Long-term exposure to neonicotinoids has negative health impacts on humans, including the disruption of the endocrine system, neurotoxic effects in children, reproductive system dysfunction, and breast cancer [4,5,6,7,8]. Additionally, IDP is resistant to biodegradation in aquatic environments and may enter water bodies through spray drift or runoff, negatively impacting algae, farmed fish [9], shrimp [10], and daphnids [3].

Consequently, it is vital to develop a simple, rapid, sensitive, and cost-effective approach for monitoring IDP residue in agricultural products and environmental samples. Traditional detection methods for IDP include high-performance liquid chromatography (HPLC) [11,12], gas chromatography–mass spectroscopy (GC-MS) [13], surface-enhanced Raman scattering (SERS) [14,15], colorimetry [16], fluorescence [17], immunoassays [18], and electrochemical methods [19]. Although immunoassays have the advantages of high specificity, sensitivity, and simplicity, they are limited by their high cost, poor stability, and the demanding conditions necessary for the preservation and chemical modification of antibodies [20,21]. While chromatographic methods provide great precision and reproducibility, electrochemical sensing offers a potential alternative to chromatography for monitoring pesticide residue due to its unmatched mobility, ability to be integrated into electronic devices, rapid measurement times, and sensitivity [22,23]. Despite these advantages, electrochemical methods tend to suffer from poor selectivity; however, the use of molecularly imprinted polymer (MIP) technology may alleviate this.

MIPs are artificial molecular receptors with a high affinity for their target molecules, and they are flexible, affordable, and thermochemically robust [24]. MIPs are functionally based on biological antibody–antigen receptor systems, wherein molecular cavities with the shape and size of an analyte of interest are formed by polymerizing monomers and crosslinkers in the presence of a template, and subsequently extracting the template molecules [25]. The molecular cavities allow for the highly specific recognition and adsorption of an analyte of interest, which is ideal for IDP detection [26]. When MIP technology is combined with electrochemical detection, the development of inexpensive and portable sensor devices is possible. In the literature, different monomer systems for imprinting IDP have been evaluated, including the conventional polyacrylate polymer system [27] and chitosan [28,29]. However, these MIP polymers impede electrochemical detection due to their electrically insulative properties. The use of MIPs designed from conducting polymers is, therefore, desirable due to their controllable electrochemical properties. Among other conductive polymers, polyaniline (PANI) is considered the most promising due to its advantages of easy preparation, good conductivity, environment stability, and tailorable electrical properties. Composite materials created from PANI and carbon materials have been found to significantly improve electrodes’ electrochemical performances compared to carbon materials alone [30].

Previously, we have reported a PANI-based molecularly imprinted Aflatoxin B1 (AFB1) microneedle (MN) sensor, which comprised the integration of carbon nanotube (CNTs) and cellulose nanocrystal (CNC) films as undercoats [26]. CNTs are excellent electrode materials due to their high electrocatalytic ability, electromechanical properties, and tailorable surface chemistry. In previous work, it was determined that the CNC, typically rich with hydroxyl groups, interacts through intra-chain hydrogen bonding with carboxylic acid-functionalized CNT to form highly stable and uniform CNC–CNT conductive films with a high electroactive area [24,26]. The CNC–CNT has a nanoporous surface with many hydroxyl groups, which is suitable for anchoring the imprinted PANI to create a stable electroactive polymer film. While, imprinted PANI have been found effective for creating molecular cavities in MIPs, the innovative use of functional monomers that can synergistically improve sensor performance remains research in progress [26,31,32,33]. We hypothesized that CNC-rich functional groups’ surface chemistry and their high surface area can be used as crosslinkers alongside PANI to increase an analyte’s selectivity for novel conductive MIPs. For the first time, we demonstrate herein an IDP-imprinted PANI-co-imidazole-functionalized cellulose nanocrystal (CNC-Im)@carbon nanotube (CNT)/CNC stainless steel MN sensor. The imidazole-functionalized CNC was prepared as described in the literature [31]. The sensor, termed MIP@CNT/CNC MN, was fabricated via layer-by-layer (LbL) assembly through the in-tube infusion of a conductive CNT/CNC composite layer, followed by an IDP-imprinted PANI-co-CNC-Im MIP polymer. The sensor, denoted MIP@CNT/CNC MN, is 4 cm in length and 0.5 mm in inner diameter and has been shown to have great potential for the electrochemical detection of IDP. In addition, the penetrative nature of the hypodermic needle sensor allows for the in-situ detection of IDP in food samples without the need for tedious sample extraction techniques.

## 2. Materials and Methods

### 2.1. Materials

The carboxylic acid functionalized-multi-walled CNTs (OD: 4–6 nm. 98% pure) were purchased from TimesNano, China. Estradiol, dopamine, potassium ferricyanide (K_3_[Fe(CN)_6_]), 3-(trimethoxyslyl)propyl methacrylate (TPM), methanol, glacial acetic acid, ammonium peroxydisulfate (APS), imidazole, sulfuric acid, silver nanoparticles (AgNPs), sodium hypochlorite, and imidacloprid (IDP) were procured from Sigma Aldrich, Ontario, Canada. Aniline, dipotassium phosphate, monopotassium phosphate, and 30% H_2_O_2_ were purchased from Fisher Scientific, USA. Cellulose nanocrystals (CNC) were donated by Alberta Innovates, Canada. Stainless steel hypodermic needles (0.7 mm × 40 mm; inner diameter 0.5 ± 0.1 mm) were purchased from a local pharmacy. All reagents were of analytical reagent grade. All aqueous solutions were prepared using >18 MΩ Milli-Q deionized (DI) water. Honey samples were purchased from Nixon Honey farm in southern Alberta, Canada.

### 2.2. Fabrication of the IDP-Imprinted PANI-co-CNC-Im@CNT/CNC MN Sensors

**Figure 1:**
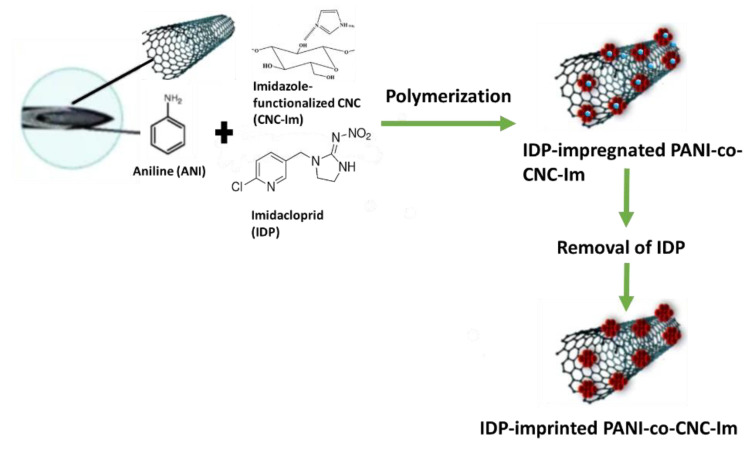
Schematic for the fabrication of the IDP-imprinted PANI/CNC@CNT/CNC MN sensor.

The MIP@CNT/CNC MN sensors were fabricated using a previous method with some modifications [34]. Briefly, the stainless MNs were immersed into piranha solution consisting of concentrated H_2_SO_4_: 30% H_2_O_2_ (1:1 *v*/*v*), which functionalized the MN surface with active hydroxyl moieties. The MNs were then silylated by immersion in an aqueous solution of TPM, DI water, and methanol in a 2:1:8 *v*/*v* ratio for 4 h. Silylation resulted in the chemical binding of organosilane moieties to the MNs’ surface. A total of 1.5 mL of a homogenous CNT/CNC (0.1%/0.4%) suspension was then infused into the silylated needle using a syringe pump at a flow rate of 15 µL/min. The CNT/CNC-modified MN was further modified with an IDP-imprinted PANI-co-CNC-Im layer. CNC-Im was produced by first sonicating 72 mg of CNC and 200 mg of imidazole in 8 mL of DI water for 2 h, and then stirring it for 24 h at room temperature. Then, a prepolymer mixture comprising 0.5 mL of 2.0 M aniline (in 1 M H_2_SO_4_), 0.5 mL of CNC-Im solution, 200 µL of 0.5 M APS initiator, and 4.0 mg of IDP template was infused into the CNT/CNC-modified needle at a rate of 15 µL/min, with the needle being incubated in an ice bath (4 °C) for polymerization to ensue. Following polymerization, the IDP template was removed via electrochemical cleaning. Briefly, the PANI-co-CNC-Im@CNT/CNC MN sensor was cleaned by 60 consecutive scans of cyclic voltammetry (CV) in 10 mL of 0.10 M PBS buffer (−1.0–1.0 V, 0.1 V/s). An IDP-imprinted PANI/CNC@CNT/CNC MN sensor fabricated using non-functionalized CNCs in the MIP layer, and a non-imprinted (NIP) PANI-co-CNC-Im@CNT/CNC MN sensor fabricated without introducing IDP template into the prepolymer solution, were utilized as controls. Figure 1 shows the schematic for the fabrication of the IDP-imprinted PANI/CNC@CNT/CNC MN sensor.

### 2.3. Instrumentation

Electrochemical measurements such as CV, differential pulse voltammetry (DPV), and electrochemical impedance spectroscopy (EIS) were carried out with a BASi Palmsens-4 potentiostat (PalmSens B.V., Houten, The Netherlands). A three-electrode system was used for all electrochemical measurements, wherein an MN electrode, platinum wire, and in-house Ag/AgCl reference electrode (composition delineated vide infra) were used as the working, counter, and reference electrodes, respectively. To fabricate the in-house Ag/AgCl reference electrode, silylated needles were first infused with a homogenous suspension comprising 1 mg/mL of CNT, 4 mg/mL of CNC, and 5 mg/mL of AgNPs at a flow rate of 15 µL/min. The CNT/CNC/AgNP-modified needles were then immersed in 0.5% NaClO bleach at room temperature overnight, and rinsed with DI water. FTIR spectra were recorded using a Bruker Tensor 27 FTIR instrument fitted with diamond-attenuated total reflectance (ATR). Scanning electron microscope (SEM) images were acquired from a Zeiss Sigma 300 VP field emission SEM. The HPLC was performed by an Agilent 1260 II Analytical-Scale LC Purification System with 6530 LC/Q-TOF system (Agilent Technologies Inc., Santa Clara, CA, USA). Separation was performed on a Superlco^®^ analytical Ascentis^®^ C18 column (Sigma-Aldrich Inc., St. Louis, MI, USA, 15 cm × 2.1 mm, 3 µM).

### 2.4. Electrochemical Measurements and Data Analysis

Both CV and DPV were employed to test the electrochemical response of the MIP@CNT/CNC MN to IDP. CV and DPV were run in the −1.0 to 1.0 V range at a scan rate of 0.1 V/s, with each scan being acquired in triplicate. The electrochemical response to IDP was determined by first measuring the blank CV/DPV in 10 mL of 0.10 M PBS buffer (pH = 7.1). Then, IDP standard (in DI water) was sequentially added, with CV/DPV being acquired after each aliquot’s addition, following 2 min of light stirring to allow for equilibration between the analyte and the sensor. The average current in the cathodic −0.38 to −0.2 V range, and the maximum peak current height within the −0.6 to −0.4 V range, were used as the analytical signals for CV or DPV, respectively. Δi (CV) was calculated by taking the difference of the average current of the sample and blank and dividing it by the blanks average current. Δi_p_ was similarly calculated by taking the difference of the maximum peak current height of the sample and blank and dividing this by the peak current of the blank. The limits of detection (LOD) were determined by three times standard deviation of the blank signal nd dividing this by the calibration sensitivity of the sensor.

The electroactive surface area of the sensors was acquired by performing CV at different scan rates ranging from 0.025 V/s to 0.50 V/s in 5 mM K_3_[Fe(CN)_6_] (in 0.10 M KCl) as a standard redox probe, and invoking the Randles–Sevcik equation [35]. Peak cathodic current (i_p_) was determined by averaging the current within the cathodic −0.39–0 V range. EIS was similarly performed in 5 mM K_3_[Fe(CN)_6_] (in 0.10 M KCl) in the frequency range of 100,000.0–0.01 Hz, with 0.01 V of sinusoidal amplitude. The electron transfer resistance (R_ct_) of each sensor was determined via circuit fitting using PSTrace 5.8 software.

### 2.5. Honey Sample Electrochemical Analysis

The MIP@CNT/CNC sensor was evaluated for the detection of IDP in a honey sample collected from the Nixon Honey Farm in Alberta. To ensure ease of use, the honey sample was first diluted with DI water in a 1:9 ratio (*w/w*). For electrochemical analysis, the standard addition method was employed, where 10 mL of 0.10 M PBS buffer was first measured as the blank. Then, 1 mL of diluted honey sample was added, followed by several aliquots of IDP standard, with CV/DPV being acquired in triplicate for each addition following 2 min of light stirring to allow for equilibration between the analyte and the sensor.

### 2.6. Honey Sample Preparation for HPLC Analysis and Settings

Both honey sample preparation and HPLC settings were adapted from [36]. Accordingly, 2.0 g of honey was dissolved in 20 mL of DI water, and then centrifuged for 5 min at 8500 rpm. Then, 5 mL of supernatant was taken and diluted in another 15 mL of DI water. This solution was then filtered through an Extract-Clean™ C18 SPE cartridge (Grace Davison Discovery Science, IL) into a clean vial for analysis. To create an external calibration curve, individual IDP standard solutions were prepared at the concentrations of 0.10, 1.0, and 5.0 mg/L in DI water.

For HPLC analysis of the honey sample and IDP standards, 0.1% folic acid in DI water (A) and acetonitrile (B) were used as the mobile phases. A gradient elution program was used, wherein the ratio of A:B varied as follows: 0 min, 10% B; 6.0 min, 70% B; 12.0 min, 70% B; 14.0 min, 10% B; 15.0 min, and 10% B, followed by a 3 min washing step at 10% B. The flow rate was 400 μL/min, the sample injection volume was 10 μL, and the temperature of the column was 30 °C. The outflow was diverted to waste from 0 to 1 min, and again from 14.5 to 15 min. The LC column effluent between 1 and 14 min was introduced into the mass spectrometer for final analysis. The mass spectrometer was operated in positive ionization mode at 350 °C and 10 L/min for the sheath gas. The nebulizer (40 psi) and drying (10 L/min) gases were both heated at 250 °C. The internal source voltage was held at 4000 V.

## 3. Results and Discussion

### 3.1. Surface Morphology

**Figure 2:**
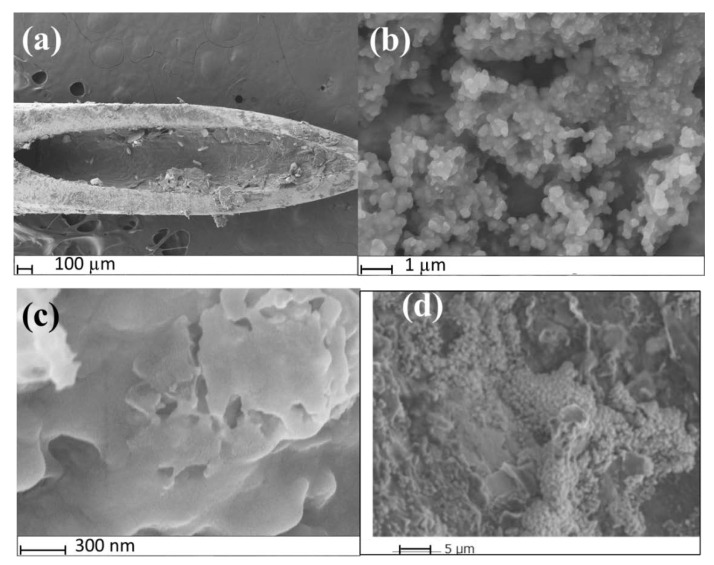
SEM images of (**a**) MIP@CNT/CNC MN sensor tip; (**b**) surface of MIP@CNT/CNC MN sensor at high magnification; (**c**) surface of the NIP@CNT/CNC at high magnification; (**d**) morphology of the surface of MIP@CNT/CNC MN sensor after repeated used.

SEM was used to confirm the successful fabrication of the MIP@CNT/CNC MN sensor. The MIP@CNT/CNC MN sensor is shown in Figure 2a, where the fibrous, crosslinked networks of the CNT/CNC composite and PANI-co-CNC-Im polymer coated on the wall of the stainless-steel MN are clearly visible [26]. Figure 2b,c represent high-magnification images of both the MIP@CNT/CNC and NIP@CNT/CNC MN sensors, respectively. Visually, the morphology of the MIP@CNT/CNC MN sensor appears more porous than the NIP@CNT/CNC MN sensor (Figure 2b,c), which is attributed to the successful imprinting of IDP in the MIP@CNT/CNC MN sensor. An analysis by ImageJ software further revealed that the pore area % was 17% for the MIP@CNT/CNC MN sensor and 3.4% for NIP@CNT/CNC MN sensor, further verifying the presence of IDP-specific cavities within the MIP@CNT/CNC MN sensor. The stability of the MIP@CNT/CNC MN sensor following repeated use was confirmed by SEM, as shown in Figure 2d.

**Figure 3:**
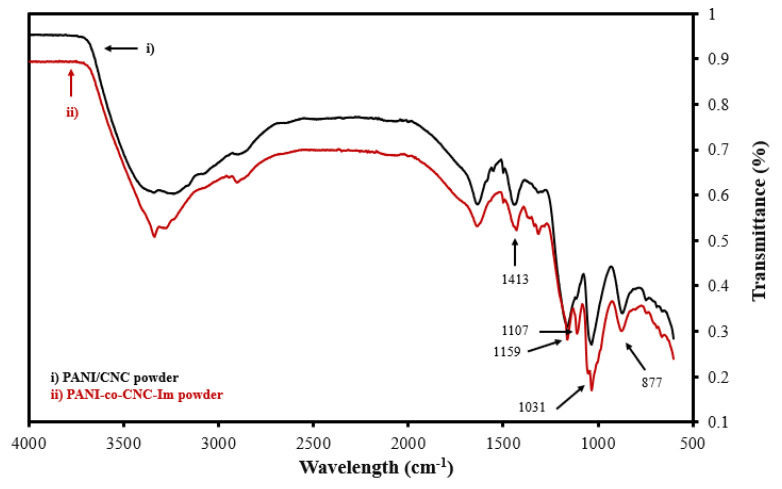
Overlaid FTIR spectra for isolated (i) PANI/CNC and (ii) PANI-co-CNC-Im polymer powders.

To verify the successful integration of CNC-Im into the PANI polymer, the functional group signatures of the isolated PANI-co-CNC-Im and PANI/CNC polymers were evaluated. Figure 3 shows the overlapped FTIR spectra of the isolated PANI-co-CNC-Im and PANI/CNC layers. The bands observed at 877, 1031, and 1159 cm^−1^ are due to the C-H deformation, C-H in-plane deformation, and C-O-C asymmetric bridging [37,38], and occur in both the PANI-co-CNC-Im and PANI/CNC polymers (Figure 2). Additionally, the band at 1413 cm^−1^ is associated with the C=C stretching vibration of the benzenoid ring in PANI [39] and is similarly found in both the PANI-co-CNC-Im and PANI/CNC polymers (Figure 3). However, the PANI-co-CNC-Im shows a unique peak at 1107 cm^−1^ (Figure 2), indicating the C-O stretching of the anhydroglucose ring linked with imidazole [37,40], confirming its presence in the PANI polymer.

### 3.2. Electrochemical Characterization

**Figure 4:**
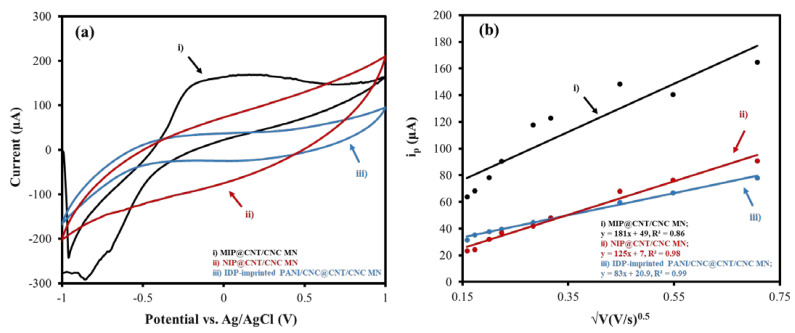
(**a**) Representative CV scans taken at 0.10 V/s and (**b**) associated peak cathodic current (i_p_) as a function of the square root of the scan rate for (i) MIP@CNT/CNC, (ii) NIP@CNT/CNC, and (iii) imidacloprid (IDP)-imprinted PANI/CNC@CNT/CNC MN sensors.

The electroactive surface areas of the MIP@CNT/CNC, NIP@CNT/CNC, and IDP-imprinted PANI/CNC@CNT/CNC MN sensors were determined via an analysis using CV at various scan rates (0.025 to 0.50 V/s) in 5 mM of K_3_FeCN_6_ (in 0.10 M KCl) and invoking the Randles–Sevcik equation [35]. Figure 4a,b illustrate the difference in the CV response and the relationship between the cathodic peak current (i_p_) and the square root of the scan rate for each MN sensor, respectively. The increased electroactive surface area between the MIP@CNT/CNC and NIP@CNT/CNC MN sensors (Table 1) is further proof of the successful formation of the IDP-shaped cavities within the PANI-co-CNC-Im polymer [26]. An increase in the electroactive surface area is also observed in both the MIP@CNT/CNC and NIP@CNT/CNC MN sensors (Table 1), indicating the enhanced electrochemical performance afforded by the PANI-co-CNC-Im polymer. 

**Figure 5:**
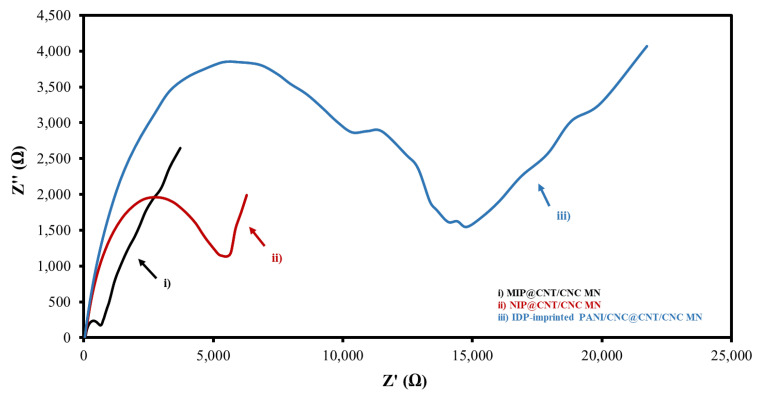
Overlapped Nyquist plot for (i) MIP@CNT/CNC, (ii) NIP@CNT/CNC, and (iii) imidacloprid (IDP)-imprinted PANI/CNC@CNT/CNC MN sensors.

An EIS analysis was also performed to determine the electrical resistances of the various IDP sensors. Figure 5 and Figure S1 show the overlaid Nyquist plots and corresponding circuit fittings for the MIP@CNT/CNC, NIP@CNT/CNC, and IDP-imprinted PANI/CNC@CNT/CNC MN sensors. Generally, a low R_ct_ is desirable for sensor conductivity and signal transduction [34]. Both the MIP@CNT/CNC and NIP@CNT/CNC MN sensors have a lower R_ct_ than the IDP-imprinted PANI/CNC@CNT/CNC MN sensor (Table 1), indicating the enhanced electrical conductivity resulting from the PANI-co-CNC-Im layer. Additionally, the enhanced electroactive surface area of the MIP@CNT/CNC sensor may also contribute to the lower R_ct_ compared to the NIP@CNT/CNC MN sensor (Table 1) [34].

### 3.3. CV Performance Evaluation

**Figure 6:**
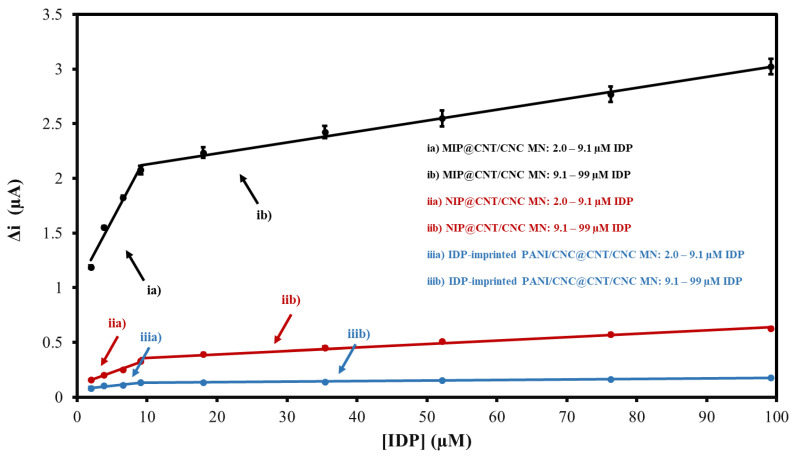
CV average current (Δi) vs. imidacloprid (IDP) concentration calibration curves for (ia) MIP@CNT/CNC (2.0–9.1 µM IDP), (ib) MIP@CNT/CNC (9.1–99 µM IDP), (iia) NIP@CNT/CNC (2.0–9.1 µM IDP), (iib) NIP@CNT/CNC (9.1–99 µM IDP), (iiia) IDP-imprinted PANI/CNC@CNT/CNC (2.0–9.1 µM IDP), and (iiib) IDP-imprinted PANI/CNC@CNT/CNC (9.1–99 µM IDP) MN sensors.

The response of the MIP@CNT/CNC MN sensor to IDP was first evaluated using CV. For the performance characterization, the response of the MIP@CNT/CNC MN sensor was compared to that of the NIP@CNT/CNC and IDP-imprinted PANI/CNC@CNT/CNC MN sensors. Figure S2 shows representative voltammograms for the three MN sensors in response to increasing IDP concentrations, with the corresponding linear profiles shown in Figure 6. In general, all sensors exhibit a higher sensitivity within the lower concentration ranges of IDP (2.0–9.1 µM) (Figure 6 and Table 2). When compared to the NIP@CNT/CNC MN sensor, the MIP@CNT/CNC MN sensor demonstrates a 4.2 × 10^2^ and 2.2 × 10^2^% increase in sensitivity in the 2.0–9.1 and 9.1–99 µM IDP concentration ranges, respectively (Table 2). Further, the imprinting factors, taken as the MIP@CNT/CNC MN sensor’s sensitivity divided by the NIP@CNT/CNC MN sensor’s sensitivity, were 5.2 and 3.2 for the lower and higher concentration ranges, respectively. This verifies the enhanced selectivity and sensitivity of the MIP@CNT/CNC MN sensor afforded by the IDP-shaped cavities in the PANI-co-CNC-Im layer. Additionally, the MIP@CNT/CNC MN sensor shows enhanced sensitivity to IDP compared to the IDP-imprinted PANI/CNC@CNT/CNC MN sensor. The sensitivity of the MIP@CNT/CNC MN sensor is 1.6 × 10^3^ and 1.9 × 10^3^% higher in the 2.0–9.1 µM and 9.1–99 µM concentration ranges, respectively (Table 2), indicating the improved IDP-sensing performance of the PANI-co-CNC-Im polymer. The percent relative standard deviation (%RSD) for the MIP@CNT/CNC MN sensor was found to be 1.9%, while the LOD was 0.35 µM.

### 3.4. DPV Performance Evaluation and Comparison to Other Works

**Figure 7:**
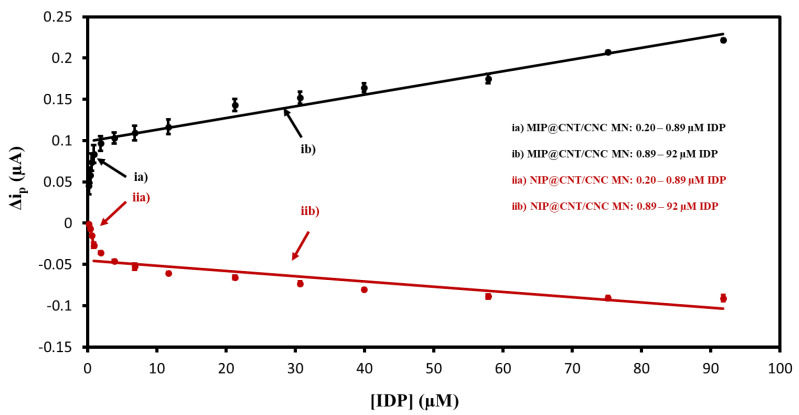
Peak DPV current (Δi_p_) vs. imidacloprid (IDP) concentration calibration curves for (ia) MIP@CNT/CNC (0.20–0.89 µM IDP), (ib) MIP@CNT/CNC (0.89–92 µM IDP), (iia) NIP@CNT/CNC (0.20–0.89 µM IDP), and (iib) NIP@CNT/CNC (0.89–92 µM IDP) MN sensors.

The MIP@CNT/CNC MN sensor was then evaluated using DPV to confirm the favorable response to IDP. Representative voltammograms are shown in Figure S3, and the corresponding linear calibration curves are shown in Figure 7. The MIP@CNT/CNC MN sensor demonstrates a 50 and 1.2 × 10^2^% increase in sensitivity in the 0.20–0.89 and 0.89–92 µM IDP concentration ranges, respectively (Table 3). The imprinting factor for the lower and higher concentration ranges was found to be 1.5 and 2.2, respectively. This validates the enhanced IDP detection performance of the MIP@CNT/CNC MN sensor over the NIP@CNT/CNC MN sensor. Lastly, the LOD for the MIP@CNT/CNC MN sensor was determined to be 0.06 µM, while the %RSD was 8.2%.

Table 4 lists the detection ranges and LODs for various electrochemical IDP sensors reported in the recent literature. The DPV method produced a lower LOD and detection range, which is preferable to the CV detection method. Additionally, these performance characteristics are superior to several of the reported sensors (Table 4).

### 3.5. Sensor Selectivity Evaluation

**Figure 8:**
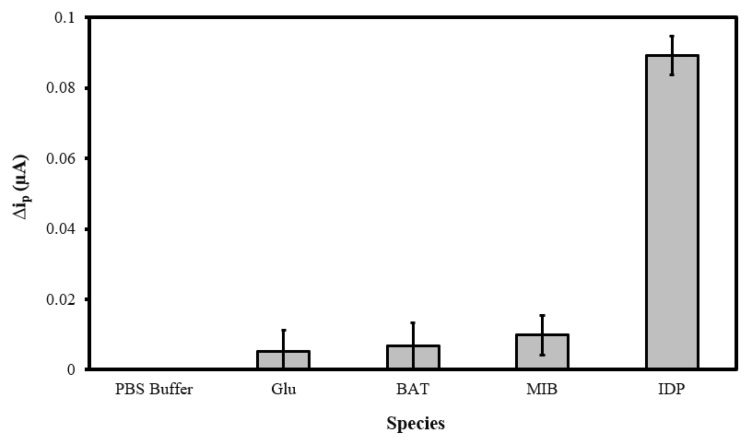
Peak DPV current (Δi_p_) obtained from sequential analysis of 0.10 M PBS blank, 1.0 µM glucose (GLU), 1.0 µM bioallethrin (BAT), 1.0 µM methyl imazamethabenz (MIB), and 1.0 µM imidacloprid (IDP) in 0.10 M PBS using the MIP@CNT/CNC MN sensor.

To verify the selectivity of the MIP@CNT/CNT MN sensor, the peak DPV currents (Δi_p_) generated in response to glucose (GLU), bioallethrin (BAT), and methyl imazamethabenz (MIB) were compared to that generated by IDP. The GLU, BAT, MIB, and IDP standards (in 0.10 M PBS) were separately analyzed, with the MIP@CNT/CNC MN sensor being allowed to equilibrate (with light stirring) for 2 min in each solution analyte prior to DPV acquisition. Representative voltammograms from the analysis of the PBS blank, 1.0 µM glucose (GLU), 1.0 µM bioallethrin (BAT), 1.0 µM Methyl imazamethabenz (MIB), and 1.0 µM imidacloprid (IDP) solutions are shown in Figure S4. The Δi_p_ generated from the analysis of the GLU, BAT, and MIB solutions are minimal compared to that generated from the IDP analysis (Figure 8). The greater Δi_p_ response verifies the selectivity of the MIP@CNT/CNC MN sensor to IDP over the other interfering analytes.

### 3.6. Honey Sample Analysis

**Figure 9:**
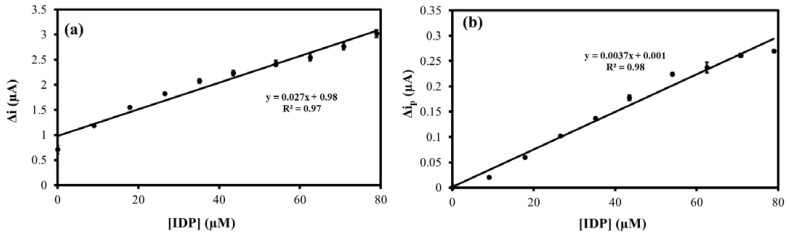
(**a**) Average CV current (Δi); and (**b**) Peak DPV current (Δi_p_) vs. imidacloprid (IDP) concentration standard addition calibration for honey sample analysis using the MIP@CNT/CNC MN sensor.

Honeybees can be exposed to IDP from the pollen of crops grown from IDP-treated seeds and pollution from the surrounding environment [46]. Thus, approximately 20% of IDP residue may remain in commercial honey products [46]. Therefore, the MIP@CNT/CNC MN sensor was evaluated for its ability to quantify IDP in a honey sample using a standard addition method. Figure S5 shows the representative voltammograms for the CV and DPV in response to the PBS blank, the honey sample, and 79 µM of IDP. The corresponding linear calibrations are shown in Figure 9. From these, the IDP concentration in the honey sample was found to be 0.9 ± 0.1 mg/g using CV, whereas the concentration found using DPV was 0.010 ± 0.004 mg/g. Using HPLC-DAD as a confirmatory method (Figure S6), the IDP concentration in the honey sample was found to be 0.013 ± 0.005 mg/g, thereby establishing the accuracy of the MIP@CNT/CNC MN sensor when using the DPV method. 

### 3.7. Sensor Reproducibility and Reusability Evaluation

**Figure 10:**
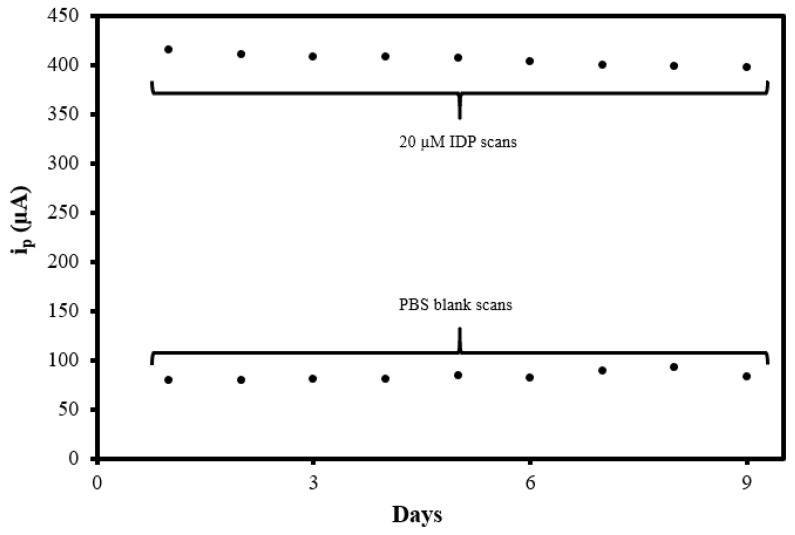
Summative peak DPV current measurement (i_p_) vs. days from the MIP@CNT/CNC MN sensor reusability test.

To investigate the reproducibility of the MIP@CNT/CNC MN sensor, the sensor was used to analyze the 0.10 M PBS blank and 20 µM IDP standard daily using DPV for a total of 9 days. The sensor was electrochemically cleaned prior to every use. As shown in Figure 10, the peak DPV current measurement (i_p_) generated from the MIP@CNT/CNC MN sensor was consistent over both the blank PBS and 20 µM IDP analysis cycles, indicative of its stability and capacity for reuse. The % RSD between the MIP@CNT/CNC MN sensor’s i_p_ signals across the nine IDP detection cycles was determined to be 1.4%. The stability of the MIP@CNT/CNC MN sensor following repeated use was confirmed by SEM, as shown in Figure 2d.

## 4. Conclusions

Imidacloprid is one of the most frequently utilized pesticides in the world and poses potential risks to both the environment and human health. This paper describes a portable, affordable, electrochemical MN sensor for the detection of IDP. Using either CV or DPV, the MIP@CNT/CNC MN sensor can detect IDP with LODs of 0.35 or 0.06 µM, respectively. Compared to recent research on IDP sensors, the reported IDP sensor is more advanced due to its wider detection range and lower LOD. In addition, the PANI@CNT/CNC MN sensor is robust and reusable and can reliably measure IDP for nine cycles over a nine-day period with high precision (% RSD = 1.4%). The PANI@CNT/CNC MN sensor can accurately determine the concentration of IDP residue within a honey sample.

## Figures and Tables

**Table 1:** Electroactive surface area and electron transfer resistance (R_ct_) values for MIP@CNT/CNC, NIP@CNT/CNC and imidacloprid (IDP)-imprinted PANI/CNC@CNT/CNC MN sensors.

Sensor Type	Electroactive Surface Area (cm^2^)	R_ct_ (kΩ)
MIP@CNT/CNC MN	0.049	0.42
NIP@CNT/CNC MN	0.034	4.5
IDP-imprinted PANI/CNC@CNT/CNC MN	0.022	9.5

**Table 2:** Linear CV calibration equations for MIP@CNT/CNC, NIP@CNT/CNC, and imidacloprid (IDP)-imprinted PANI/CNC@CNT/CNC MN sensors in response to IDP.

Sensor Type	2.0–9.1 µM CV Linear Equation	9.1–99 µM CV Linear Equation
MIP@CNT/CNC MN	y = 0.12x + 1.02 R^2^ = 0.97	y = 0.0100x + 2.03 R^2^ = 0.99
NIP@CNT/CNC MN	y = 0.023x + 0.11 R^2^ = 0.99	y = 0.0032x + 0.33 R^2^ = 0.98
IDP-imprinted PANI/CNC@CNT/CNC MN	y = 0.007x + 0.072 R^2^ = 0.94	y = 0.00051x + 0.127 R^2^ = 0.96

**Table 3:** Linear DPV calibration equations for MIP@CNT/CNC and NIP@CNT/CNC MN sensors in response to imidacloprid (IDP).

Sensor Type	0.20–0.89 µM DPV Linear Equation	0.89–92 µM DPV Linear Equation
MIP@CNT/CNC MN	y = 0.056x + 0.036 R^2^ = 0.97	y = 0.00142x + 0.099 R^2^ = 0.96
NIP@CNT/CNC MN	y = −0.038x + 0.007 R^2^ = 0.99	y = −0.0006x−0.045 R^2^ = 0.81

**Table 4:** Literature comparison of imidacloprid (IDP) sensor performance metrics to the reported MIP@CNT/CNC MN sensor.

Sensor Name	Detection Range (µM)	LOD (µM)	Reference
**PoPD at reduced graphene oxide (RGO) modified electrode**	0.75–70	0.40	[41]
**MAA-EGDMA MIP sensor**	5–100	4.61	[42]
**Nitrogen-doped graphene (NGE)**	5–100	0.55	[43]
***f*** **MWCNT-Nafion^®^_0.5%/_GCE**	0.2–1.77	0.0374	[19]
**RPC@PANI/GCE**	0.400–274	0.117	[44]
**Fe_3_O_4_@SiO_2_@MIPIL fluorescent sensor**	0.001–0.01	0.0003	[45]
**IDP-imprinted PANI-co-CNC-Im@CNT/CNC MN sensor**	2.0–99 (CV) 0.20–92 (DPV)	0.35 (CV) 0.06 (DPV)	This work

## Data Availability

All relevant data is included in the manuscript and in the supporting information.

## Supplementary Materials

The following supporting information can be downloaded at: https://www.mdpi.com/article/10.3390/s22218492/s1, Figure S1: EIS circuit fittings for the different IDP sensors; Figure S2: Overlaid CVs (0.10 V/s) obtained from the analysis of 0.10 M PBS blank, 2.0, 9.1, and 99 µM imidacloprid (IDP) using the (a) MIP@CNT/CNC; (b) NIP@CNT/CNC; and (c) IDP-imprinted PANI/CNC@CNT/CNC MN sensors; Figure S3: Overlaid DPVs (0.10 V/s) obtained from the analysis of 0.10 M PBS blank, 0.20, 0.89, and 92 µM imidacloprid (IDP) using the (a) MIP@CNT/CNC and (b) NIP@CNT/CNC MN sensors; Figure S4: Overlaid DPVs (0.10 V/s) obtained from the sequential analysis of 0.10 M PBS blank, 1.0 µM glucose (GLU), 1.0 µM bioallethrin (BAT), 1.0 µM methyl imazamethabenz (MIB), and 1.0 µM imidacloprid (IDP) in 0.10 PBS using the MIP@CNT/CNC MN sensor; Figure S5; Overlaid (a) CVs (0.10 V/s) and (b) DPVs (0.10 V/s) obtained from the analysis of 0.10 M PBS blank, honey sample, and 79 µM imidacloprid (IDP) using the MIP@CNT/CNC MN sensor; Figure S6: Overlaid LC-MS chromatograms (@ 270.4 nm) obtained from a honey sample, 0.10, 1.0, and 5.0 mg/L imidacloprid (IDP) standards; and (b) peak signal intensity vs. IDP concentration external calibration curve.
