# Supplementary material for: Molecularly Imprinted Polymer-Modified Microneedle Sensor for the Detection of Imidacloprid Pesticides in Food Samples
Source: Sensors (Basel). 2022 Nov 4;22(21):8492. doi: 10.3390/s22218492 (PMC9655949; doi:10.3390/s22218492)
Supplement: Supplementary file 1 [file sensors-22-08492-s001.zip › sensors-1917311-supplementary.pdf]

Molecularly Imprinted Polymer-Modified Microneedle Sensor for the Detection of Imidacloprid Pesticides in Food Samples

Samuel M. Mugo *, Weihao Lu and Scott V. Robertson

Department of Physical Sciences, MacEwan University, Edmonton, AB T5J4S2, Canada
* Correspondence: mugos@macewan.ca

Supporting Information

Figure S1. EIS circuit fittings for the different IDP sensors.

a) MIP@CNT/CNC MN sensor

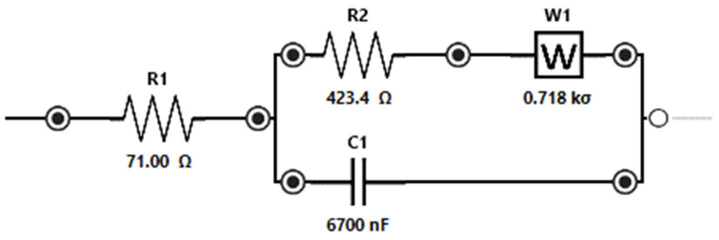

b) NIP@CNT/CNC MN sensor

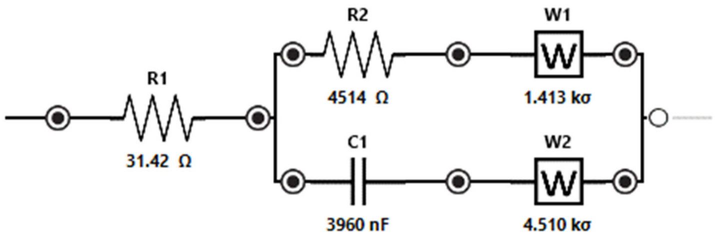

c) IDP-imprinted PANI/CNC@CNT/CNC MN sensor

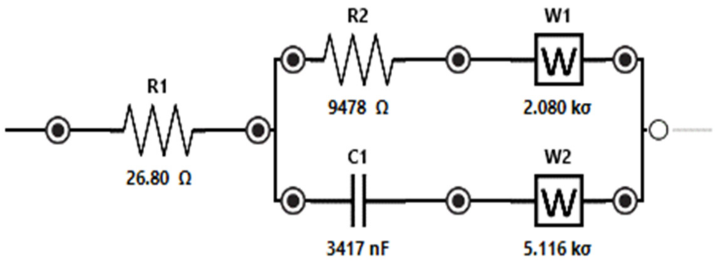

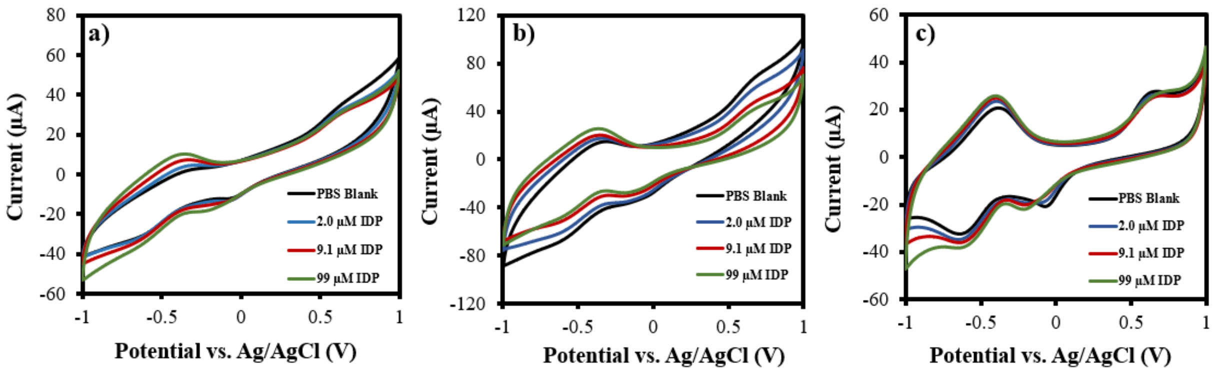

Figure S2. Overlaid CVs (0.10 V/s) obtained from the analysis of 0.10 M PBS blank, 2.0, 9.1, and 99 µM imidacloprid (IDP) using the **a)** MIP@CNT/CNC; **b)** NIP@CNT/CNC; and **c)** IDP-imprinted PANI/CNC@CNT/CNC MN sensors.

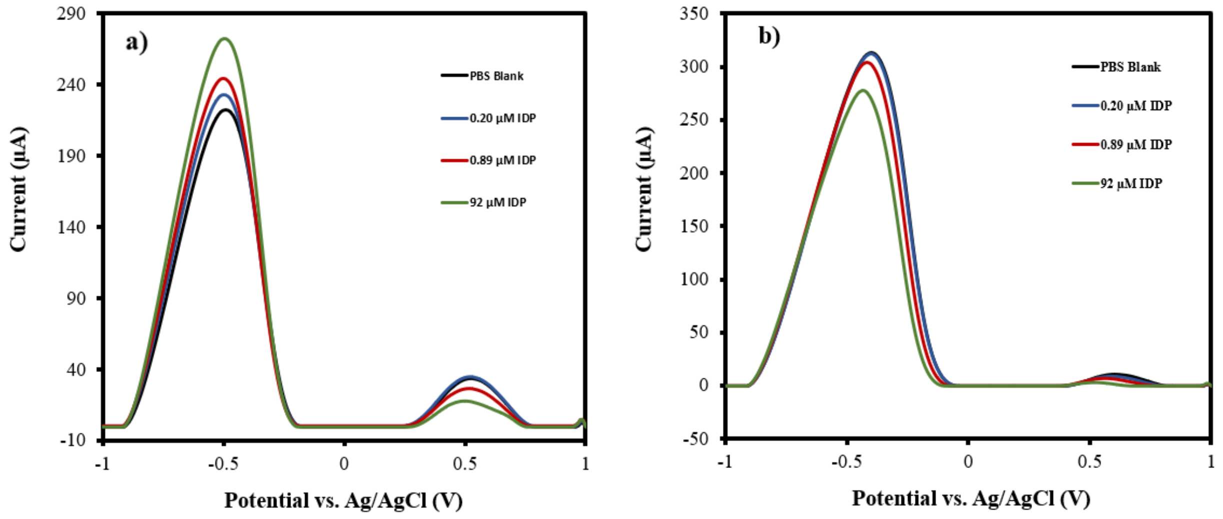

Figure S3. Overlaid DPVs (0.10 V/s) obtained from the analysis of 0.10 M PBS blank, 0.20, 0.89, and 92 µM imidacloprid (IDP) using the **a)** MIP@CNT/CNC and **b)** NIP@CNT/CNC MN sensors.

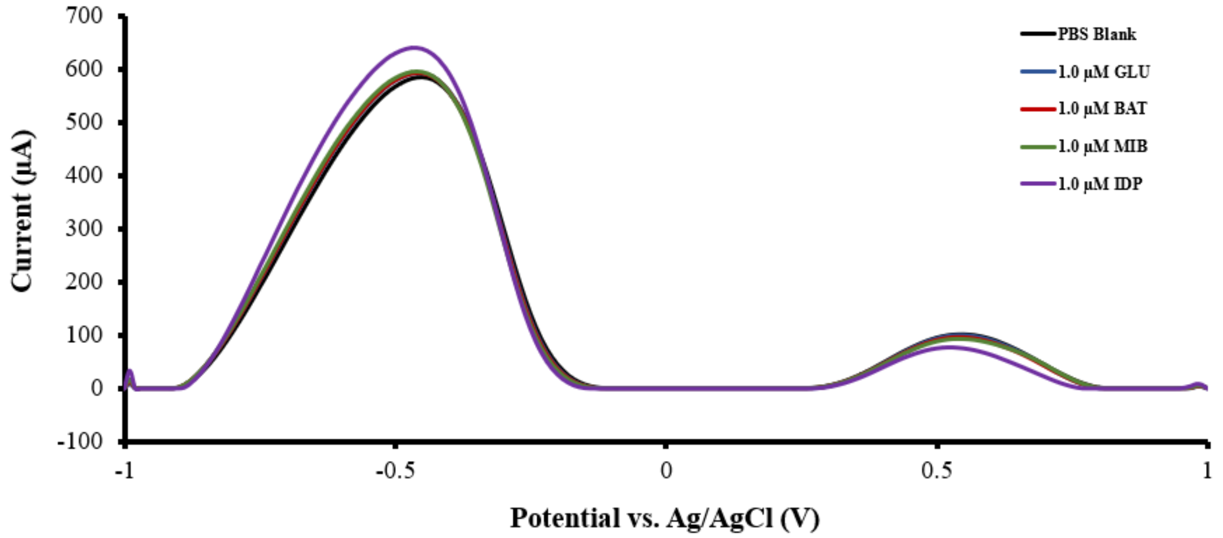

Figure S4. Overlaid DPVs (0.10 V/s) obtained from the sequential analysis of 0.10 M PBS blank, 1.0 µM glucose (GLU), 1.0 µM bioallethrin (BAT), 1.0 µM methyl imazamethabenz (MIB), and 1.0 µM imidacloprid (IDP) in 0.10 PBS using the MIP@CNT/CNC MN sensor.

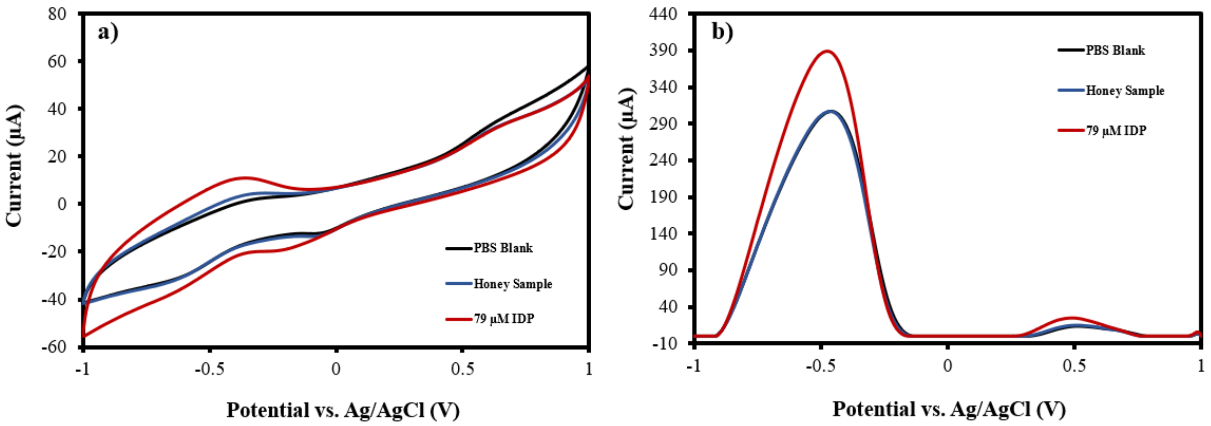

Figure S5. Overlaid **a)** CVs (0.10 V/s) and **b)** DPVs (0.10 V/s) obtained from the analysis of 0.10 M PBS blank, honey sample, and 79 µM imidacloprid (IDP) using the MIP@CNT/CNC MN sensor.

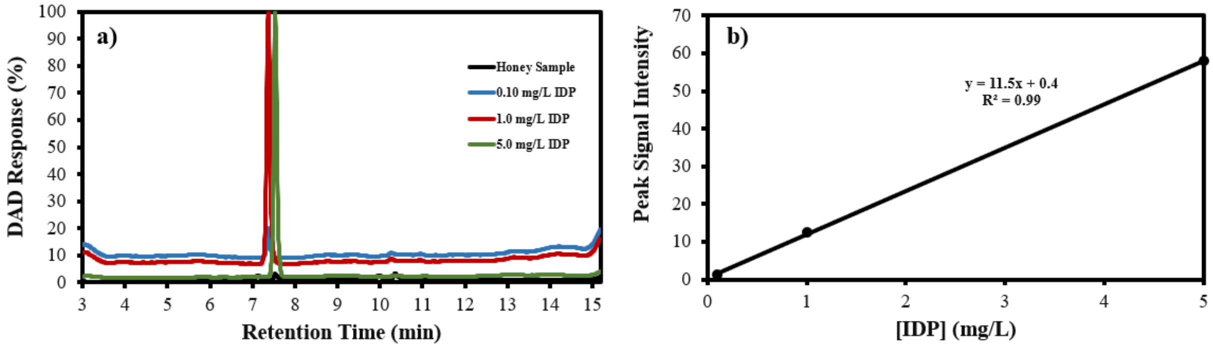

Figure S6. a) Overlaid LC-MS chromatograms (@ 270.4 nm) obtained from a honey sample, 0.10, 1.0, and 5.0 mg/L imidacloprid (IDP) standards; and **b)** peak signal intensity vs. IDP concentration external calibration curve.
